# The future of veterinary communication: Partnership or persuasion? A qualitative investigation of veterinary communication in the pursuit of client behaviour change

**DOI:** 10.1371/journal.pone.0171380

**Published:** 2017-03-03

**Authors:** Alison M. Bard, David C. J. Main, Anne M. Haase, Helen R. Whay, Emma J. Roe, Kristen K. Reyher

**Affiliations:** 1 School of Veterinary Sciences, The University of Bristol, Bristol, United Kingdom; 2 School for Policy Studies, The University of Bristol, Bristol, United Kingdom; 3 Geography and Environment, University of Southampton, Southampton, United Kingdom; University of British Columbia, CANADA

## Abstract

Client behaviour change is at the heart of veterinary practice, where promoting animal health and welfare is often synonymous with engaging clients in animal management practices. In the medical realm, extensive research points to the link between practitioner communication and patient behavioural outcomes, suggesting that the veterinary industry could benefit from a deeper understanding of veterinarian communication and its effects on client motivation. Whilst extensive studies have quantified language components typical of the veterinary consultation, the literature is lacking in-depth qualitative analysis in this context. The objective of this study was to address this deficit, and offer new critical insight into veterinary communication strategies in the pursuit of client behaviour change. Role-play interactions (n = 15) between UK cattle veterinarians and an actress experienced in medical and veterinary education were recorded, transcribed and analysed thematically. Analysis revealed that, overall, veterinarians tend to communicate in a directive style (minimal eliciting of client opinion, dominating the consultation agenda, prioritising instrumental support), reflecting a paternalistic role in the consultation interaction. Given this finding, recommendations for progress in the veterinary industry are made; namely, the integration of evidence-based medical communication methodologies into clinical training. Use of these types of methodologies may facilitate the adoption of more mutualistic, relationship-centred communication in veterinary practice, supporting core psychological elements of client motivation and resultant behaviour change.

## Introduction

The protection of animal health and welfare is central to the veterinarian identity, conveyed and embedded via their oath upon admission to the Royal College of Veterinary Surgeons; ‘*I promise… that*, *above all*, *my constant endeavour will be to ensure the health and welfare of animals committed to my care’* [1]. Fulfilling this oath is complex, requiring not only the scientific expertise on animal health gained via training in veterinary science, but the ability to effectively communicate this expertise to animal owners to encourage its implementation through behaviour change (whether by administering treatments, enacting management processes, or a multitude of other actions). Communication training has received increased emphasis over the last decade, with all UK veterinary institutions now teaching the Calgary-Cambridge model to enhance clinical communication skill and improve client outcomes [2]. However, in practice, veterinarians still struggle with the dual role of scientific advisor and proactive communicator [3], evidenced by low rates of adherence with veterinary recommendations in many areas [4]. For example, little change has been seen in the prevalence of lame dairy cattle in decades, despite extensive scientific research on risk factors and management strategies implicit in their occurrence enhancing veterinary advice [5].

Research suggests that the typical veterinarian communication style stems from the relationship dynamic established between veterinarian and client. In veterinary consultations, the predominant approach is that of paternalism, where the veterinarian sets the consultation agenda, takes on the role of the guardian and assumes that the client’s values match their own, resulting in veterinarians contributing most of the talking and clients playing a passive role [6]. This ensures veterinary communication is largely directive in style; for example veterinarians use predominantly closed questions, rarely employ empathetic statements in relationship building and rarely encourage client participation in appointments [7–9].

Despite the intuitive appeal of this persuasive style based on assumptions of efficiency [10], it is more likely to elicit client reactions against a behaviour rather than in favour of it (a phenomenon known as psychological reactance [11]) due to the ambivalence clients commonly experience in the contemplation of change. This directive approach also offers little opportunity to meet the basic psychological needs necessary for inspiring motivation: that of autonomy (volition over behaviour), relatedness (to experience connection with another) and competence (perceived self-efficacy) [12]. The predominance of this consultation approach, combined with its conflict with basic motivational principles, may contribute to why uptake of veterinary recommendations are reported as low in a wide range of settings [4].

Awareness of this issue is already taking hold in the veterinary profession. In a recent consultation with veterinarians, veterinary nurses and clients, the Vet Futures project [13] established a need for a ‘paradigm shift’ from this *‘hierarchical model with the vet as the expert imparting instruction*, *to one centred on partnership with empowered clients and other veterinary-related professionals’*. This reflects a professional shift away from paternalism towards mutuality, a relationship-centred approach where client opinions are actively sought and open negotiation leads to a mutually agreed upon plan [6, 14]. This may offer important improvements in the uptake of veterinary advice, for studies of relationship-centred care in the medical profession have demonstrated a positive relationship to physician and patient satisfaction [15], patient health outcomes [16] and reduced complaints of malpractice [17]. To support this shift, it is essential to have a detailed understanding of the language and communication strategies currently used in veterinary consultations, to ensure recommendations for communication improvements are meaningfully targeted. Current data have quantified language components typical of the veterinary consultation [6–8, 18, 19], however, in depth qualitative analysis of the mechanisms by which this is achieved within a consultation are not recorded.

The aim of this research was to identify strategies commonly employed by veterinarians in communication with the aim of behaviour change. For this purpose, role-play interactions were selected to ensure that communication strategies employed were a function of veterinarian approach, not client variation in response; one role-play actress was used for all veterinarian-client interactions. To reflect an appropriate context in the veterinary realm in which the complexities of communication, client ambivalence and behaviour change are witnessed, the context of advisory services on cattle lameness and mastitis were selected. These diseases are endemic in the UK dairy industry [20, 21] and have seen little change in recent decades [5, 21]. Veterinarians are also known to struggle with communication and proactive advice [3], exacerbated by farmer ambivalence stemming from the myriad complexities of herd health management [22]. The focus of our study was driven by two research questions: (1) what consultation strategies are prominent in communication with the aim of behaviour change and (2) how do veterinarians attend to client motivation, understanding and engagement with advice when communicating with the aim of behaviour change.

## Materials and methods

Role-play sessions reflecting consultations on lameness and mastitis were recorded between cattle veterinarians (n = 15) recruited from two UK practices located in South West England and an actress experienced in role-play scenarios in both medical and veterinary education. Consultations were held in a closed room at the workplace of each practice with only the veterinarian, actress and researcher (Bard) present, and were recorded via an Olympus DS-3500 digital voice recorder. Each practice engaged in one session of data collection, between February and March 2015.

The actress was not provided with a script, or cues of any kind, for the purpose of this interaction. Instead, she was provided with a character and farm profile reflecting a ‘typical’ UK situation, indicating mean herd size, productivity, lameness and mastitis levels. Background information on the farmer’s family, perceived barriers to uptake of advice and attitudes/norms/perceived control of lameness and mastitis were also provided. The actress then improvised during each interaction, responding to the communication received in an appropriate and genuine manner given this profile, as a means to generate authentic simulation of the veterinarian-client encounter.

During each ‘consultation’, veterinarians were provided with a short excerpt on the disease issue on the farm, an indication of the risk factors that were likely to be involved, and evidence to encourage them to broach a broad topic area of change with the farmer. For lameness, the broad topic was early detection and treatment of lame cows; for mastitis, it was use of the AHDB Dairy Mastitis Control Plan [23]. Veterinarians were given their script at the start of their session; data collection commenced when they stated they had had enough time to consider it and had asked any relevant questions. Veterinarians were limited to fifteen minutes for the interaction, and were informed of this; if this time limit approached, the actress would improvise a natural closing of the interaction. The role-play scenario was piloted with a cattle veterinarian from the University of Bristol in advance of data collection; data was not recorded from this pilot for inclusion in the study.

### Participants

In summary (Table 1), the veterinarians in this study were an average age of 37 years (range 24 to 54) and had been in practice an average of 15 years (range 3 to 29). The majority (13/15) had experience in general/mixed practice. Veterinarians were a convenience sample, recruited by email, telephone or face-to-face interactions from practices known to the research team. Not all veterinarians within each practice chose to participate, due to conflict between practice obligations and timing of data collection. For anonymity purposes, the number of participating veterinarians from each practice is not included in this paper.

**Table 1:** Participant demographics for veterinarians (n = 15) in role-play interactions.

Demographic	Veterinarians
Gender	Male (9)
	Female (6)
Age in years	21–30 (6)
	31–40 (3)
	41–50 (3)
	51–60 (3)
Years in practice as a veterinarian	1–5 (5)
	6–10 (2)
	11–15 (3)
	16–20 (2)
	21 + (3)

### Analysis

The 15 role-play interactions were transcribed (*verbatim*) by external transcribers for analysis. Transcripts and audio were initially explored using traditional paper-based coding methods, allowing assessment of the data and the development of initial coding ideas. Data were then imported into the qualitative software NVivo 10 (QSR International) for thematic analysis [24]. All textual analysis was supported by listening to audio data in conjunction with transcript analysis. The entire dataset was coded using inductive themes (i.e. themes determined by the data set and not *a priori*). This resulted in a hierarchical coding structure of three core themes, with various subthemes attributed to each core concept. Once complete, a sub-sample of participants (n = 4) were provided with the study results to receive feedback, which supported the authenticity of the work.

### Research team

Research was primarily carried out by one female researcher (Bard) undertaking a PhD in Clinical Veterinary Science at the University of Bristol, with a background in Animal Behaviour and Welfare Science (BSc) and training in qualitative research methodologies. Coding was cross-examined by one female supervisor (Roe), an experienced social and cultural geographer.

### Ethics statement

This study was reviewed and approved by the University of Bristol Research Ethics Committee, ensuring procedures met ethical guidelines in place for research with human participants. An information sheet was supplied to participants detailing the aims of research prior to data collection, with written consent to take part obtained both before initiating and after completing each role-play interaction. Participants were aware that the study was focused on communication and the uptake of veterinary advice.

## Results and discussion

**Fig 1:**
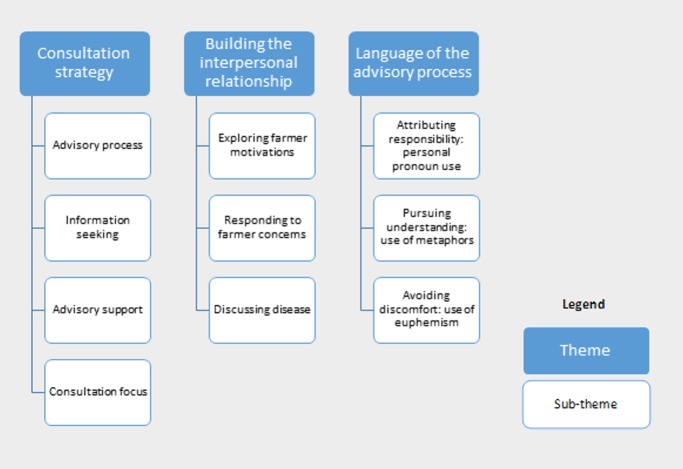
Themes and subthemes identified by thematic analysis of role-play (n = 15) discourse.

Consultations lasted an average of 11.9 minutes (range 7.7 to 14.9). Thematic analysis revealed three prominent themes as summarised in Fig 1: Firstly, the *language of the advisory process*, encompassing the effects of verbal framing of both disease and control mechanisms; secondly, the *consultation strategy*, where typical veterinarian approaches to shaping advisory discourse emerged; thirdly, *building the interpersonal relationship*, reflecting interactions underpinning how the veterinarian-farmer relationship was established.

### Theme 1. Consultation strategy

#### 1.1. Advisory process

**Fig 2:**
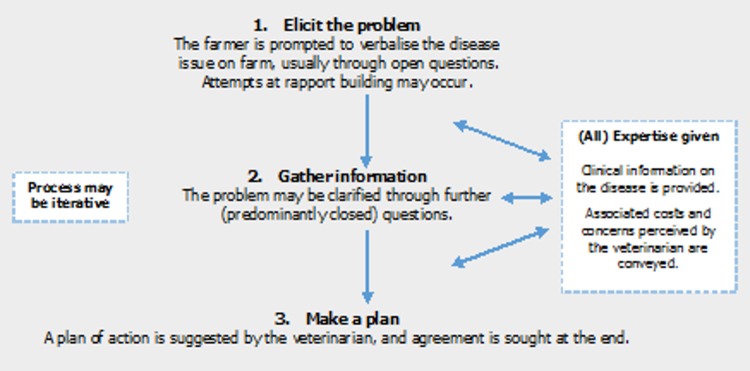
Three core consultation stages identified by thematic analysis of role-play (n = 15) discourse. Veterinarians generally utilised open questions at Stage 1, enquiring how the farmer felt about current issues.

In all role-plays, veterinarian dialogue on lameness and mastitis had a common, overarching strategy. This can be presented at its simplest as Fig 2.

RP 11 Veterinarian

“Right, so how are things going on the farm?”

**Fig 3:**
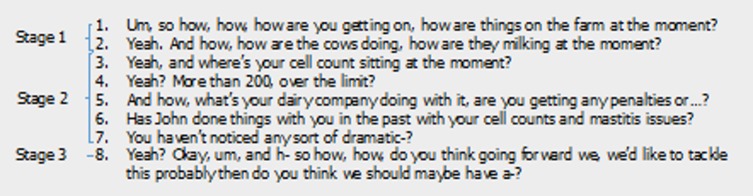
Veterinarian language illustrating the three consultation stages identified by thematic analysis of role-play (n = 15) discourse. Language represents all veterinarian questions in the first 20% of consultation time (Role-Play 5).

In Stage 2 clarification of the issue was sought through further—predominantly closed—questions. In Stage 3, concrete statements were made on what action should be taken by the ‘farmer’, making a plan for moving forward. For example, Fig 3 represents questions used in the first 20% of interaction time in Role-Play 5. Questions move from Stages 1–3, first eliciting the problem, then clarifying the issue and finally, moving on to planning.

These consultation steps emerged through each interaction, albeit with variation in the time veterinarians allocated to each step and the number of iterations of the whole process. Most frequently, veterinarians focused fleetingly on Stage 1, then moved repeatedly back and forth between Stages 2 and 3, constantly clarifying aspects of the problem and using this to deliver additional information that linked back to an ultimate plan/rationale. In a minority of cases, veterinarians concentrated on eliciting much more information from the ‘farmer’ first, spending a considerable amount of time in Stages 1 and 2 before broaching Stage 3.

#### 1.2. Information seeking

When seeking information, veterinarians demonstrated a strong preference for the use of closed questions, with on average four closed questions asked for every one open question. Question types were associated with the consultation stages previously described, with Stage 1 (elicit) relying on open questions such as:

RP 2 Veterinarian

“What are your thoughts on the lameness levels at the moment on the farm?”

and Stage 2 (gather information) relying predominantly on closed questions (Fig 3).

#### 1.3. Advisory support

In support of their advisory recommendations, veterinarians relied on a four core topics:

(1) The evidence base or research associated with advice:

RP 3 Veterinarian

“But it’s interesting that there’s some **more work and papers of research** coming out which suggest that there are slightly different ways of focusing on it”

(2) The experiences of other farmers:

RP 4 Veterinarian

“And the other thing we can do is, um, have a chat with **some of the other guys in our practice** who are currently already using this. And you might well find that, that, er, what they’ve got to say is quite encouraging as well.”

(3) the veterinary profession (themselves, the veterinary practice, and veterinarian construct as a whole):

RP 5

Veterinarian

“Yes, well there’s, there’s plenty of people around that we can, you know, that we can use to help us, so I don’t have to do it all on my own and we can, we can use other people if, if necessary”

Farmer

“What other people are you talking about?”

Veterinarian

“Other people in the practice.”

and (4) external influencers (such as milk buyers):

RP 14 Veterinarian

“Yes, I don’t know who your milk buyer is, but some of the **milk buyers** it is something that they’re wanting to see records for, and it might increase in future that other milk buyers do”

#### 1.4. Consultation focus

Overall, the ‘focus’ of the consultation was dominated by the veterinarian. All veterinarians created a conversation focused on, and largely limited to, immediate factors surrounding the disease process as identified in the scenario information. That is, little emphasis was placed on asking the ‘farmer’ about wider issues, attitudes or ideas, or allowing ‘farmer’ comments to divert the conversation away from disease management. In questions, this was achieved by a focus on fact-finding questions that supported the veterinarian’s interest, constituting, on average, four out of five queries:

RP 11 Veterinarian

“So what’s your bulk milk somatic cell count at the moment?”

RP 5 Veterinarian

“So how are the cows doing, how are they milking at the moment?”

Client perspective questions—those aimed at eliciting the thoughts and feelings of the ‘farmer’—on average constituted less than one question in every six:

RP 12 Veterinarian

“So were you pleased with how we got on with the routine this morning?”

RP 5 Veterinarian

“How do you think going forward we’d like to tackle this probably then…?”

In non-questioning veterinarian speech, veterinarian focus on disease advice was maintained by taking steps to actively direct the conversation towards planning and goal setting. This was achieved by combining a quick succession of ‘disease facts’ (disease risks and costs) with a ‘solution statement’ (how a plan of action would solve these), thereby minimising the opportunity for opposing arguments:

RP 6 Veterinarian

[1] Fact establishment [2] solution statement

“[1]Cause you’ve got two....cause from the point of view of the cow, if you can get her foot lifted and treated as soon as she goes lame, you’ll probably have her back right again in no time and she’ll be much more profitable animal to use. [2] It definitely pays you to treat her straight away; the question probably is whether or not you get somebody in to do it or whether you’re happy to do it yourself.”

This process appeared in two forms: a concise form (as above), where the disease facts and solution statement follow one another in a single statement, or an expanded form, where the veterinarian would guide the ‘farmer’ through disease facts in a series of questions and statements, to conclude with a solution statement(s). The latter process often occurred iteratively throughout consultations (data not shown).

### Theme 2. Building the interpersonal relationship

#### 2.1. Exploring farmer motivations

Through all 15 role-play interactions, the ‘farmer’ was not asked directly about her values, goals or motivations. Reference to motivation was only made once, indirectly, following discussion of breeding replacement heifers to improve the herd age distribution:

RP 15 Veterinarian

“If it’s something you’ve highlighted already and something that you’re **motivated** to do then obviously that’ll be something that definitely I can help you work towards.”

Six veterinarians used open-ended questions aimed at eliciting the concerns of the farmer:

RP 12 Veterinarian

“Yeah. What’s… what’s.....what’s worrying you most at the moment?”

This acted as a functional equivalent: by eliciting the ‘farmer’s’ concerns and opening a discussion on the issues worrying her, the veterinarian was able to open a (possible) route to exploring where or why she might be motivated to make a change. However, for the majority of veterinarians, the ‘farmer’s’ motivation is implicitly assumed, not explicitly sought, throughout interactions. Instead, veterinarians used ‘typical’ motivators to underpin their advice, such as monetary cost, input of time and improvement of yields:

RP 11 Veterinarian

“I can put some figures and stuff together for you as well to sort of indicate where your benefits and stuff are going to … and the-the … basically the dollar value is the–is the key thing isn’t it?”

#### 2.2. Responding to farmer concerns

When responding to a concern expressed by the farmer, veterinarians typically showed instrumental support—offering tangible help and solutions—by indicating practical support mechanisms:

RP 7

Farmer

“Right, yeah I get what you’re saying. I do worry about the money side of things and that’s not your problem, that’s mine.”

Veterinarian

“Well there are....occasionally there are funded schemes that come in for these sort of things, which can be really useful and I don’t think there’s one going at the moment, but we recently had this big um SWHLI lameness project where you get....you get funding”

Or offering a ‘solution’ statement which inferred that the concern raised could be dealt with:

RP 13

Farmer

“I don’t know if I’ve got the time available to do anything else, because we are so limited. You know, we’ve got two small kids as well”

Veterinarian

“It may not mean doing more. It may just mean… it may just mean doing different. So, you know, it may be that we can, for example, alter or suggest alterations to the milking routine which actually don’t take any long....any longer. It may even be quicker, but which would reduce the risk of mastitis spreading within the herd”

However, explicit emotional support—attending to and exploring the client’s perspective or feelings and communicating an understanding thereof—was rarely employed in advisory dialogue. Only two veterinarians used complex reflective statements during their interactions, clarifying and restating what the ‘farmer’ conveyed to encourage further exploration:

RP 12

Farmer

“Yeah. But....so it..... I’m not saying..... I think what you’re saying is very good. I’m just thinking in my head “Oh my God!” [laughs]”

Veterinarian

“It’s.... it’s one other thing that I’m trying to get you to do on top of all the other things that I’m trying to get you to do with mastitis and that sort of thing as well. So it does become a bit..... a bit overwhelming.”

#### 2.3. Discussing disease

One strategy employed was to emphasise the normality of disease on farm:

RP 1 Veterinarian

“Well to be honest that’s the, you know, you, **you’re not alone**, so don’t feel bad about that, there’s plenty of farmers with that.”

RP 3 Veterinarian

“Wh- what’s, what’s the main problem out there at the minute? How are you, how are you getting on with the, the **usual difficulties** in the farming industry?”

### Theme 3. Language of the advisory process

#### 3.1. Attributing responsibility: Personal pronoun use

Throughout the role-play, veterinarians varied their pronoun use greatly. In gathering information about the farm and generating farmer opinion, use of the second person singular ‘you’ predominated (typical of conversational speech where ‘you’ takes the place of a noun to address an individual):

RP 1 Veterinarian

“How often are **you** scraping?”

This pronoun was also used when referring to current farm ‘problems’ such as high mastitis levels:

RP 2 Veterinarian

“Well I think, I think what we need to do to start with is to, is to work out what those cows that are a problem at the moment, just to sort of get a diagnosis on those cows, and then as time goes on, hopefully **you** will get less and less new cows.”

When discussing plans of action for the herd, or recommendations for changes to practice, veterinarians would employ the inclusive first person plural ‘we’, indicating themselves and the farmer as the subjects of speech:

RP 1 Veterinarian

“So it’s really important to look at the whole picture, and what **we’d** need to do is- the first thing **we** did before **we** did anything is look at your records, and just try and work out exactly where the problem is.”

This was incongruent with farmer language over management actions; all management-related thoughts expressed by the ‘farmer’ in these role-plays were presented in the first person singular ‘I’.

The first person plural ‘we’ was also utilised as an exclusive term denoting themselves and someone external to the farmer/conversation, such as the veterinary practice:

RP 9 Veterinarian

“So there’s....there’s a couple of things that....that we’ve started doing as a....as a practice if you like, cause **we’re** quite....**we’re** quite keen on the old…on the old lameness”

Or sometimes the ‘we’ is more ambiguous, and merely seems to reflect ‘myself and the veterinary profession’:

RP 2 Veterinarian

“**We** now know, and there’s good research to back this up, to show that they’re much more likely to get better quicker and they’re also less likely to go lame again in the future. Okay?”

#### 3.2. Pursuing understanding: Use of metaphor

Metaphors were used to simplify understanding of disease processes:

RP 12 Veterinarian

“Most of the time it’s um…… sole ulcers are like um..... a good way to think of them is like, you know, if you um, er, if you cut your**..... if you squeezed your finger in a vice** and you’ve got some bleeding under your nail, it’s**.... it’s that sort of thing except the vice in this case is cows standing on concrete for too long.”**

To convey an understanding of the challenges farmers encounter in the management of disease:

RP 9 Veterinarian

“You’re not really very different to any, you know other farmer in the area, but if you start wherever you are and sort of think ‘oh we could be....have no lame cows at all’, **that’s just a mountain** and it’s....it’s not achievable at the end of the day.”

and to convey the ideal disease management process:

RP 9 Veterinarian

“Yeah, yeah and they find that, you know, how these things are all kind of inter-related, the fertility and the lameness and the mastitis and all the rest of it, and **if you can chip away at one corner of that kind of, you know, pyramid,** you can kind of improve…improve the whole thing.”

The strength of the former metaphor for this process was seen when it was mirrored by the ‘farmer’ when querying the benefits of early detection and treatment of lameness:

RP 9 Farmer

“.....getting on top of anything sooner is better than later than, but how does that affect the yield? Cause you were **talking about this pyramid** and knock on effect and all of that?”

#### 3.3. Avoiding discomfort: Use of euphemism

Disagreement aversion was witnessed in the descriptive terminology of lameness, as illustrated by opening statements on the issue. Some veterinarians employed a ‘softer’ approach, not using the word lameness itself, but instead inferring the issue using more informal, euphemistic terms:

RP 9 Veterinarian

“I did....did just sort of spot moving through a couple…couple of those girls, sort of taking....**taking their time** to get....get into the race there. Have you had a sort of few girls **lagging behind**, getting into the parlour, that kind of thing?”

In contrast, some brought up the issue more directly under the clinical term:

RP 6 Veterinarian

“She’s very **lame** isn’t she? What’s…what’s the matter with her?”

## Discussion

**Fig 4:**
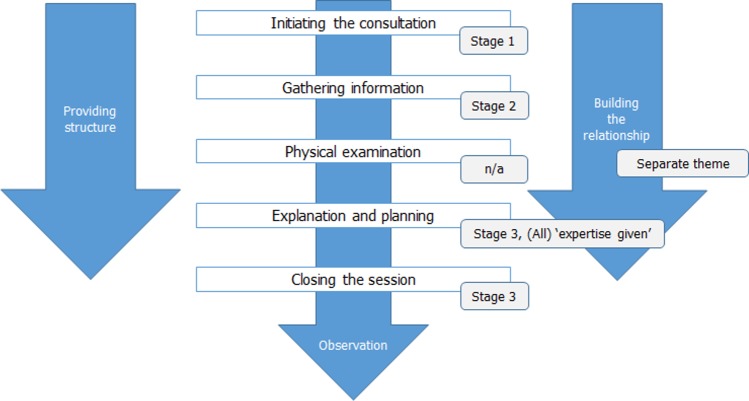
Congruence of consultation stages identified by thematic analysis of role-play (n = 15) discourse and Calgary-Cambridge model. Adapted from [26] for illustrative purposes.

The aim of this research was to identify strategies commonly employed by veterinarians in communication with the aim of behaviour change, driven by two research questions; (1) what consultation strategies are prominent in communication with the aim of behaviour change and (2) how do veterinarians attend to client motivation, understanding and engagement with advice when communicating with the aim of behaviour change. Overall, qualitative analysis of role-play data supports existing quantitative analysis of veterinary communication. The emergent consultation process resonates with the core elements of the Calgary-Cambridge model, posited by Silverman et al. [25] (Fig 4) and widely adopted in the veterinary realm [2]. In small animal consultations, these iterations and structure are also witnessed [19], indicating that the model either reflects something critical about standard veterinary communication processes, or standard communication processes have been influenced by the widespread teaching and distribution of the model. Communication behaviours additionally reflect those witnessed in wider literature [7–9]; veterinarians dominated the agenda, typically placed minimal value on eliciting the client’s own motivations and ideas within a consultation, kept strictly to the topic of disease management at the expense of rapport building and prioritised instrumental support strategies.

It is possible to hypothesise that the cause of these behaviours is rooted in the methodology. A limitation of this study is that the role-plays were artificial; veterinarians were time limited (>15 minutes), did not have an established relationship with the client and were ‘performing’ a role. However, these features are not unrepresentative of wider research reflecting ‘naturally occurring’ consultations. Mean role-play consultation length (11.9 minutes) is certainly comparable to small animal practice [6, 18, 19], whilst advisory interactions on-farm are often restricted to fit around other practical tasks, despite longer contact time with clients (for example, interspersing cattle fertility checks). In naturally occurring consultation data, where relationships are established and no ‘performance’ is occurring, both directive behaviours [7–9] and a dearth of emotional support [7, 8] are still witnessed. Whilst a limitation of the study, the methodology alone can therefore not account for the strategies that emerged. We hypothesise that these strategies were witnessed as a result of the relational paradigm of paternalism recognised in veterinary literature [6], as our data reflect characteristics implicit in this style: the professional sets the consultation agenda, communicates in a directive style and contributes most of the talking, leaving the client in a passive role.

Indeed, the language of attributing responsibility suggests that this paternalistic approach may be heavily integrated into the veterinary identity, to the extent of shaping pronoun use when discussing disease management processes. Veterinarians relied on the collaborative pronoun ‘we’ for discussing management actions on farm, yet the ambiguity of this term undermined any assumptions of collaborative intent; it is impossible to determine whether veterinarians were or were not fostering partnership, or whether the ‘farmer’ did or did not perceive this. What is measurable in these data is the incongruence of this pronoun with all ‘farmer’ language on the same topic of management; all management-related thoughts expressed by the ‘farmer’ were presented in the first person singular ‘I’, suggesting these were actions she alone—not the veterinarian—would have to take. This pronoun incongruence and advisor reliance on ‘we’ to initiate action statements is also witnessed in doctor-patient exchanges. It is speculated to reflect a situation where doctors retain the right to direct the agenda; the term ‘we’ may act as a vehicle for directive discourse by which topics are nominated for discussion. The incongruence of doctor-client pronoun use infers that doctors are viewed as conduits or coordinators of, but not participants in, care [27]. Our veterinarian-farmer incongruence may similarly indicate this nuance within the consultation.

These paternalistic strategies are likely to impact on client motivation to engage in behaviour change. A strictly veterinarian directed consultation focus reduced client choice and opportunity for self-direction within the consultation, thwarting a sense of autonomy [12]. The dominance of instrumental support and deficit in explicit emotional support created an interaction where the client was likely to feel less empathised with [28], impeding relatedness. Minimal opportunities for the client to vocalise and explore their ability, intention and rational for change(s) diminished a sense of self-efficacy in the planning process. Where these factors are critical to inspire motivation and the internalisation of behaviour [12] the conflict between this paternalistic style and psychological attributes is significant. As these communication strategies are reflected in the wider veterinary literature [7–9], this conflict may underpin issues with adherence to recommendations and behaviour change in the veterinary setting.

However, despite this paternalistic approach, veterinarians are able to concurrently employ strategies in these consultations that enhance a sense of relatedness. Euphemism was used to avoid discomfort over recommendations and discussions of disease, for example substituting the term ‘lameness’ with ‘lagging behind’. By using euphemism in social interaction, communicators either seek to minimise potential discomfort in an addressee [29] or, more frequently, use this language as self-preservation to appear more sympathetic or considerate [30]. Metaphor use also supported the building of rapport by generating a shared understanding of advisory recommendations; veterinarians shaped and strengthened farmer perception of advice by evoking a host of multiple meanings [31]. The dominance of instrumental support (tangible help/solutions) may also reflect an attempt by veterinarians to display empathy, rather than a lack thereof; veterinarians may be perceiving the farmer’s concern as a negative emotional state and trying to alleviate it by providing a ‘role appropriate’ response (‘fixing’ the problem, as they are paid to do). Previous literature suggests that this behaviour readily occurs in professional interactions, where advisor support strategies are shaped by their focus on alleviating a problem [28]. If done skilfully, this instrumental verbal behaviour is likely to carry symbolic emotional meaning for a receiver [28], positively influencing relatedness. It is, of course, impossible to determine the veterinarian’s intention, or whether the ‘farmer’ attributed emotional significance to this strategy. However, previous literature suggest the ‘farmer’ is less likely to feel emotionally engaged with when instrumental support is used, compared to when she receives overt emotional support [28].

Veterinarians also show a complex understanding of motivational factors underpinning farmer decision making through varied advisory support strategies, despite the absence of overt evocation or consideration of the ‘farmer’s personal opinion. Citing research may reflect the move towards evidence-based veterinary medicine (EBVM), and the responsibility to *‘ground … decisions on sound*, *objective and up-to-date evidence*, *when available’* [32]. When referring to other farmers, veterinarians display an intuitive understanding of the psychological components of change, recognising that personal perceptions of other people’s behaviour (subjective norms) exert influence over the intention to change one’s own behaviour [33]. When citing the support of the veterinary profession, veterinarians are conveying a notion of their professional status and authority as a part of this unit, cultivating the interpersonal trust that is critical to the uptake of advice [34]. Finally, aligning recommendations with future economic incentives (milk price) reflects awareness of economic issues facing the dairy industry that may be exerting great pressure on farmers; market volatility is certainly of great concern [35]. Overall, these strategies tell us that the typical veterinarian is balancing a complex set of approaches in what is easily reduced to ‘directive advice’. Their awareness of farmer psychology, changes within the profession and challenges to the farmer are all captured within their approach; what is missing is attending to the client’s perspective to actively tailor this communication to the individual, rather than responding with generalities.

These qualitative data therefore provide an optimistic view of the future of the veterinary consultation. Whilst they confirm communication deficits in empathy, collaboration and motivation as recognised in existing literature [7, 8], the results presented here suggest that veterinarians may already be motivated to create an environment that meets these needs. Unfortunately, the paternalistic role of the veterinarian—an expert, paid to provide a service of advice and solutions—may shape these responses into the directive language and structure with which they are delivered. As discussed earlier, this style creates psychological reactance [11], so, ironically, the very service this professional style aims to deliver is directly counteracted by the communication strategies it produces.

In light of these data, the paradigm shift towards mutuality in the future of veterinary services becomes more complex. To promote client motivation and behaviour change within veterinary consultations, is it simply enough to ask for more partnership when the subtle effects of the existing paternalistic paradigm are likely to undermine it? This conflict is well illustrated via the Vet Futures report [13] which states that ‘*by working* ***in partnership*** *with clients*, *vets are* ***better positioned to convince them*** *of the value of preventive services’*. The conflict between mutuality and paternalism here is clear: to ‘convince’ is the essence of paternalism, suggesting the need to bring another to our point of view, to direct their opinions and choices. As a result, the alluded partnership is merely presented as a vehicle to better persuade in a directive style, rather than an approach in its own right. To stimulate a genuine paradigm shift, future communication training may need to incorporate methodologies that foster a mutualistic approach as the *backbone* of practice rather than a useful aid. For example, one such evidence-based methodology widely adopted in the medical and psychological sciences is Motivational Interviewing (MI). MI practice is not just defined by a set of verbal skills cultivating empathy, collaboration and support of patient autonomy, but by an underpinning philosophy of compassion, acceptance, partnership and evoking (eliciting client ideas, rather than imposing) that act as a mindset to guide practice [36]. Familiarity with communication philosophies of this type, and appropriate implementation in veterinary consultations within the wide remit of models like the Calgary-Cambridge, would offer novel insights to veterinarians in practice into how communication of these important motivational factors could best be achieved.

## Future work

This paper explores the complex nature of veterinary advice for farmer behaviour change, using role-play as a means to explore current communication strategies. This methodology was chosen to control for variation in ‘client’ response during the interactions, given that role-play provides *‘a variety of naturally occurring data and therefore worthy of study’*[37], yet the potential for role-play to generate ‘authentic simulations’ is a complex issue [38] and may be considered a limitation of this research. Future work could address this matter with the analysis of naturally occurring data (i.e. unrecorded, routine veterinarian-client interactions of this nature) to investigate if the same themes emerge given varied animal health topics and the complexity of differing clients and environments. Additionally, widening the veterinary context these data are taken from to include naturally occurring farm, small animal and equine consultations would add additional strength to the thematic process.

The collection and analysis of naturally occurring data would also ensure that the underlying complexity in ‘real world’ encounters is represented; the human-animal relationship. The socially constructed categories of ‘companion’ and ‘livestock’ animals engender differing human perceptions and practices [39] affecting both the owner-animal and veterinarian-animal relationship, in addition to veterinarian perceptions of their client’s relationship to their animal (and thus perceived motivation to attend to health and welfare). Investigating across the boundaries of farm, small animal and equine services would illuminate the applicability of these findings given this variation. However, as the trends in these data mirror those found in communication research in companion animal practice (such as veterinarian dominance in agenda setting, minimal solicitation of client opinion and lack of explicit emotional support [7, 8]), the data presented here already appear to represent something meaningful about veterinary communication across these contexts.
